# Metabolic Syndrome: Is Arthroscopic Rotator Cuff Repair Safe in This Patient Population?

**DOI:** 10.7759/cureus.39299

**Published:** 2023-05-21

**Authors:** Alana Sadur, Theodore Quan, Chelsea Nguyen, Sean Tabaie

**Affiliations:** 1 Orthopaedic Surgery, George Washington University School of Medicine and Health Sciences, Washington DC, USA; 2 Orthopaedic Surgery, Children's National Hospital, Washington DC, USA

**Keywords:** surgery, outcomes, metabolic syndrome, complications, arthroscopic rotator cuff repair

## Abstract

Purpose

Metabolic syndrome is associated with postoperative morbidity and mortality in surgical patients. With the increased use of arthroscopic techniques for rotator cuff repair (RCR), it is important to identify the impact this disorder has on surgical patients. The purpose of this study is to evaluate the clinical impact of metabolic syndrome on outcomes following arthroscopic RCR.

Methods

The 2006-2019 National Surgical Quality Improvement Program database was queried for adult patients who underwent arthroscopic RCR. Two patient groups were categorized: patients with metabolic syndrome and patients without metabolic syndrome. Demographics, comorbidities, and 30-day postoperative outcomes were compared using bivariate and multivariate analyses.

Results

Of 40,156 patients undergoing arthroscopic RCR, 36,391 did not have metabolic syndrome and 3,765 had metabolic syndrome. After adjusting for differences in baseline characteristics between the two groups, those with metabolic syndrome had an increased risk of developing renal complications and cardiac complications, as well as requiring hospital admission postoperatively and hospital readmission.

Conclusion

Metabolic syndrome is an independent risk factor for developing renal and cardiac complications, as well as requiring overnight hospital admission and hospital readmission. Providers should understand the need for preoperative evaluation and surveillance of these patients following their surgery to minimize the risk of poor outcomes.

## Introduction

Metabolic syndrome is defined by the National Cholesterol Education Program’s Adult Treatment Panel III criteria as the presence of at least three or more of the following risk factors for cardiovascular disease: high fasting blood glucose (>100 mg/dL), low levels of high-density lipoprotein (<40 mg/dL for men and <50 mg/dL for women), high levels of serum triglycerides (>150 mg/dL), large waist circumference (>40 inches for men, >35 inches for women), high blood pressure (>130/85 mmHg), and use of medications for any of the aforementioned conditions [1]. The presence of metabolic syndrome put patients at a significant risk of developing serious cardiovascular conditions including heart disease, diabetes, and stroke. Given the aging population in the United States, the increase in prevalence of metabolic syndrome in the aging population is of utmost concern. Although several risk factors are known to contribute to metabolic syndrome, the main underlying risk factors are obesity and insulin resistance [2]. Physical inactivity has also been found to further exacerbate this condition. This is exceedingly important as there is a significant prevalence of physical inactivity in the United States [3]. While several studies found that sedentary lifestyle, defined by accelerometer, has differing results related to metabolic syndrome, many studies have shown an association between physical inactivity and metabolic syndrome [4].

Prior studies have evaluated the association between metabolic syndrome and postoperative complications following orthopedic surgeries. Cheng et al. found a four-fold increased risk of postoperative wound infection following total hip arthroplasty in patients with metabolic syndrome [5]. A systematic review and meta-analysis by Guofeng et al. found that metabolic syndrome was associated with a higher risk of cardiovascular complications, surgical site infections, urinary tract infections, and 30-day readmission following total hip and total knee arthroplasty [6]. Marigi et al. found that while patients with metabolic syndrome did not have a significantly increased risk of postoperative complications, infection, r-operation, or revision following shoulder arthroplasty, patients with metabolic syndrome were found to have significantly higher rates of complications following reverse shoulder arthroplasty [7].

Arthroscopic rotator cuff repair (RCR) has been the preferred approach compared to open repair for several reasons. Day et al. found higher rates of total 30-day complications including surgical infections in patients undergoing open RCR compared to arthroscopic repair [8]. A similar study by Baker et al. found that patients who underwent open approach had an increased risk of adverse events, return to the operating room within 30 days, and longer hospital stay [9]. Studies have shown that patients with metabolic syndrome may be at a higher risk of shoulder pain and rotator cuff tears in addition to an association between low-grade inflammatory biomarkers and rotator cuff-related shoulder pain [10].

Despite the significant amount of literature detailing the increased risk of morbidity, mortality, and postoperative complications in patients with metabolic syndrome, there is minimal research evaluating postoperative complications in patients following arthroscopic RCR. Considering this lack of research, combined with the increased use of arthroscopic technique for RCR, this study aims to evaluate the clinical impact of metabolic syndrome on patients undergoing this operation.

## Materials and methods

The study design was a retrospective cohort study using a national database. IRB approval was not required for the study as the database is publicly available and all patient data have been de-identified. Patient data were obtained from the American College of Surgeons National Surgical Quality Improvement Program (ACS-NSQIP) database. The study period was from 2006 to 2019. This national database is a multi-institutional registry that includes patient data from more than 600 participating hospitals [11]. This database is a risk-adjusted, outcome-based quality improvement program whose goal is for the improvement of surgical outcomes. All data are validated by trained reviewers through the use of medical records, patient interviews, and operative reports, with a very low disagreement rate [12]. Variables collected include preoperative, intraoperative, and postoperative data [13].

Patient selection

The patient population undergoing arthroscopic RCR was identified using Current Procedural Terminology (CPT) code 29827. Patients with missing baseline demographics data were excluded from the study. Patients were also excluded if they were younger than 18 years or if they had disseminated cancer, sepsis, or wound infection on admission. Patients who had another open shoulder procedure performed concomitantly with their arthroscopic RCR were also excluded. The study cohort was categorized into two groups: patients with metabolic syndrome and patients without metabolic syndrome. Based on previous studies, metabolic syndrome was defined by a predetermined criterion, which consists of the simultaneous presence of diabetes mellitus, hypertension, and body mass index (BMI) > 30 kg/m^2^ [14,15].

Patient characteristics

Baseline demographic and clinical characteristics of the study population included age, gender, race, American Society of Anesthesiologists (ASA) classification, smoking status, and functional status. The study population’s comorbidities collected included chronic obstructive pulmonary disease (COPD), congestive heart failure (CHF), requirement for dialysis, chronic steroid usage, bleeding disorder, and dyspnea. The type of anesthesia used during the surgery was also recorded.

Thirty-day outcomes

Patients were followed for 30 days postoperatively to see if they experienced any complications. These complications were categorized into clinically relevant domains. These domains included renal (renal failure or renal insufficiency), pulmonary (pneumonia, reintubation, or failure to wean off ventilator for greater than 48 hours), cardiac (cardiac arrest or myocardial infarction), sepsis (sepsis or septic shock), wound (superficial surgical site infection, deep surgical site infection, organ space infections, or wound dehiscence), thromboembolic (deep vein thrombosis, stroke, or pulmonary embolism) complications. Other 30-day complications assessed were postoperative transfusion, urinary tract infection (UTI), hospital admission postoperatively, extended length of stay, readmission, reoperation, and mortality. Based on previous studies, hospital admission postoperatively was defined as at least one overnight stay in the hospital immediately after the operation, whereas extended length of stay was defined as hospital stay of more than two days [16,17].

Statistical analysis

The Statistical Package for the Social Sciences (SPSS, Version 26, IBM Corp., Armonk, NY) software was used for the different statistical tests conducted in this study. Bivariate analyses were used to compare the baseline demographics and comorbidities between the metabolic syndrome cohort and the non-metabolic syndrome cohort. Chi-squared tests and analysis of variance were used where appropriate. Postoperative outcome data were also analyzed between the two cohorts. Demographic and comorbidity variables were included in the multivariate regression models for p-values < 0.20 to control for covariates [18]. A p-value of <0.05 was the cut-off for significance in this study.

## Results

Demographics

After application of the exclusion criteria, 40,156 patients undergoing arthroscopic RCR were included in the analysis. Of these patients, 36,391 patients (90.6%) did not have metabolic syndrome and 3,765 (9.4%) had metabolic syndrome. In the metabolic syndrome cohort, the average BMI was 37.21 kg/m^2^. When compared to the patients without metabolic syndrome, those with metabolic syndrome were more likely to be older, female, and Black or African American, have an ASA classification of III or IV, and have a dependent functional status (p<0.001 for all). On the other hand, patients without metabolic syndrome were more likely to be smokers (p<0.001) (Table 1).

**Table 1:** Baseline demographics and clinical characteristics for arthroscopic rotator cuff repair patients ¶Pearson’s chi-squared test. **Analysis of variance. ASA, American Society of Anesthesiologists; SD, standard deviation

Demographics	No Metabolic Syndrome	Metabolic Syndrome	P-value
Total patients, n	36,391	3,765	
Gender, n (%)			<0.001^¶^
Female	15,326 (42.1)	1,699 (45.1)	
Male	21,065 (57.9)	2,066 (54.9)	
Race, n (%)			<0.001^¶^
White	28,350 (77.9)	2,635 (70.0)	
Black or African American	3,220 (8.8)	562 (14.9)	
Hispanic	3,320 (9.1)	440 (11.7)	
Native American or Alaska Native	250 (0.7)	36 (1.0)	
Asian	1,085 (3.0)	69 (1.8)	
Native Hawaiian or Pacific Islander	166 (0.5)	23 (0.6)	
ASA, n (%)			<0.001^¶^
I or II	25,337 (69.6)	907 (24.1)	
III or IV	11,054 (30.4)	2,858 (75.9)	
Smoker, n (%)	5,486 (15.1)	478 (12.7)	<0.001^¶^
Dependent functional status, n (%)	145 (0.4)	37 (1.0)	<0.001^¶^
Mean age, years (SD)	58.28 (11.03)	61.13 (8.64)	<0.001**

Comorbidities

In comparison to patients without metabolic syndrome, those with metabolic syndrome were more likely to have other medical comorbidities, including COPD, CHF, dialysis requirement, bleeding disorder, and dyspnea on moderate exertion (p<0.01 for all) (Table 2).

**Table 2:** Comorbidity and intraoperative data for arthroscopic rotator cuff repair patients ¶Pearson’s chi-squared test COPD, chronic obstructive pulmonary disease; CHF, congestive heart failure; MAC, monitored anesthetic care

Comorbidities	No Metabolic Syndrome	Metabolic Syndrome	P-value^ ¶^
Total patients, n	36,391	3,765	
COPD, n (%)	1,047 (2.9)	199 (5.3)	<0.001
CHF, n (%)	40 (0.1)	13 (0.3)	<0.001
Dialysis, n (%)	40 (0.1)	12 (0.3)	0.001
Chronic steroid use, n (%)	710 (2.0)	90 (2.4)	0.066
Bleeding disorder, n (%)	503 (1.4)	122 (3.2)	<0.001
Dyspnea, n (%)			<0.001
Moderate exertion	984 (2.7)	246 (6.5)	
At rest	43 (0.1)	15 (0.4)	
Anesthesia type, n (%)			0.035
General	33,506 (92.5)	3,526 (93.8)	
Neuraxial	58 (0.2)	7 (0.2)	
Regional	1,445 (4.0)	123 (3.3)	
MAC	996 (2.7)	79 (2.1)	

Complications

On bivariate analysis, relative to patients without metabolic syndrome, those with metabolic syndrome were more likely to develop renal, pulmonary, and cardiac complications (p<0.01 for all). Metabolic syndrome patients were also more likely to be admitted to the hospital postoperatively, have an extended length of stay, be readmitted to the hospital, and experience mortality (p<0.001 for all) (Table 3).

**Table 3:** Bivariate analysis of postoperative outcomes for arthroscopic rotator cuff repair patients ¶Pearson’s chi-squared test

Thirty-Day Complications	No Metabolic Syndrome	Metabolic Syndrome	P-value^¶^
Total patients, n	36,391	3,765	
Renal complication, n (%)	5 (0.0)	3 (0.1)	0.006
Pulmonary complication, n (%)	55 (0.2)	15 (0.4)	0.001
Cardiac complication, n (%)	20 (0.1)	12 (0.3)	<0.001
Sepsis complication, n (%)	18 (0.0)	0 (0.0)	0.172
Wound complication, n (%)	63 (0.2)	9 (0.2)	0.363
Thromboembolic complication, n (%)	111 (0.3)	11 (0.3)	0.891
Postoperative transfusion, n (%)	6 (0.0)	2 (0.1)	0.129
Urinary tract infection, n (%)	72 (0.2)	8 (0.2)	0.848
Hospital admission postoperatively, n (%)	2,549 (7.0)	459 (12.2)	<0.001
Extended length of stay, n (%)	553 (1.5)	104 (2.8)	<0.001
Readmission, n (%)	328 (1.2)	68 (2.3)	<0.001
Reoperation, n (%)	99 (0.3)	15 (0.4)	0.165
Death, n (%)	5 (0.0)	4 (0.1)	<0.001

Following adjustment on multivariate regression models to control for the differences in baseline characteristics between the two cohorts, compared to patients without metabolic syndrome, those who had metabolic syndrome had an increased risk of developing renal complications (OR: 6.30; 95% CI: 1.32 to 30.10; p=0.021) and cardiac complications (OR: 4.10; 95% CI: 1.89 to 8.89; p<0.001), requiring hospital admission postoperatively (OR: 1.37; 95% CI: 1.23 to 1.54; p<0.001), and also being readmitted to the hospital within 30 days (OR: 1.35; 95% CI: 1.02 to 1.78; p=0.035) (Table 4). 

**Table 4:** Multivariate regression analysis of postoperative outcomes for arthroscopic rotator cuff repair patients CI, confidence interval.

Metabolic Syndrome (Versus No Metabolic Syndrome)	Odds Ratio	95% CI		P-Value
Renal complication	6.297	1.317	30.096	0.021
Pulmonary complication	1.768	0.960	3.255	0.067
Cardiac complication	4.096	1.886	8.894	< 0.001
Hospital admission postoperatively	1.374	1.228	1.539	< 0.001
Extended length of stay	1.158	0.923	1.453	0.205
Readmission	1.348	1.021	1.780	0.035
Death	3.501	0.876	13.992	0.076

## Discussion

While metabolic syndrome has been extensively studied in the literature, the risk of complications following arthroscopic RCR in patients with metabolic syndrome has not been studied to our knowledge. There have, however, been several studies comparing complications in patients with metabolic syndrome following other orthopedic procedures. Several studies have found an association between orthopedic procedures and adverse outcomes for patients with metabolic syndrome [19]. The results of this study are consistent with several other studies, which found that metabolic syndrome was associated with an increased risk of postoperative complications. In a study by Guofeng et al., the authors found that patients with metabolic syndrome who underwent total hip or total knee arthroplasty were at an increased risk of postoperative complications of all causes and 30-day hospital readmission [6]. A similar study by Edelstein et al. found that metabolic syndrome was an independent risk factor for Centers for Medicare and Medicaid Services reportable conditions, wound complications, and readmission after total hip and total knee arthroplasty [20]. Tracy et al. determined that metabolic syndrome was a risk factor for postoperative complications, longer length of stay, and increased risk of readmission in patients following orthopedic trauma surgery [21]. Furthermore, for patients undergoing reverse shoulder arthroplasty, Marigi et al. found that patients with metabolic syndrome were at an increased risk of deep infections compared to those without metabolic syndrome [7].

The impact of metabolic syndrome has also been investigated in spinal surgeries. Following elective lumbar fusion, Chung et al. indicated that metabolic syndrome was a predictor of acute renal failure; however, this condition only slightly increased the risk of overall complications and was not found to be associated with unplanned reoperation or readmission [15]. In the geriatric population, a study found that metabolic syndrome was associated with postoperative complications including progressive renal insufficiency, acute renal failure, urinary tract infection, and readmission following various hip fracture surgeries [22]. However, the results from our study and the aforementioned studies are different from several other studies, which found no association between metabolic syndrome and postoperative outcomes. Malik et al. found that metabolic syndrome was not associated with 30-day complications, reoperations, or readmissions in patients following anterior cervical discectomy and fusions [23]. A similar study found that metabolic syndrome was not associated with postoperative complications or extended length of stay following total shoulder arthroplasty [24].

Nevertheless, given the findings of the present study and prior studies, it is of utmost importance to be aware of the potential for an increased risk of postoperative complications in patients with metabolic syndrome who undergo arthroscopic RCR. A possible reason for these postoperative complications is the increased comorbidities, which exist in patients with metabolic syndrome. In addition to having hypertension, diabetes mellitus, and BMI > 30 kg/m^2^, which defines metabolic syndrome, patients in this study also had other comorbidities including COPD, CHF, dialysis requirement, bleeding disorder, and dyspnea on exertion. Although we controlled for these other comorbidities on multivariate analysis, it is possible that these patients had further comorbidities, which were not captured by the database and therefore were not able to be accounted for which may have influenced the results. There were several other limitations to the current study. The NSQIP database only records patient data for up to 30 days following the surgery; therefore, only short-term complications were able to be elucidated. Future studies should examine longer-term complications, such as 90-day, one-year, or two-year complications. Also, as with all database studies, this study may have errors in data omission or erroneous data entry. Furthermore, the NSQIP database does not include data on patient-reported outcomes, such as quality-of-life scores or functional scores. Future studies should evaluate these outcomes in metabolic syndrome patients undergoing arthroscopic RCR.

Patients with metabolic syndrome are at an increased risk of renal and cardiac complications, postoperative hospital admission, and unplanned hospital readmission within 30 days following arthroscopic RCR compared to those without this condition.

## Conclusions

With the increased adoption of sedentary lifestyles and physical inactivity in addition to the increasing prevalence of metabolic syndrome with age, it is important to be aware of the increased risk of complications in patients with metabolic syndrome following arthroscopic RCR so that appropriate pre- and postoperative care of these patients is performed. This study aimed to evaluate the impact of metabolic syndrome on various outcomes following arthroscopic RCR. The results of this study showed that following arthroscopic RCR, patients with metabolic syndrome had an increased risk of developing renal and cardiac complications and they were more likely to require hospital admission postoperatively as well as be readmitted to the hospital within 30 days of their surgery.
